# Effectiveness of Home Visits in Pregnancy as a Public Health Measure to Improve Birth Outcomes

**DOI:** 10.1371/journal.pone.0137307

**Published:** 2015-09-08

**Authors:** Kayoko Ichikawa, Takeo Fujiwara, Takeo Nakayama

**Affiliations:** 1 Department of Health Informatics, Kyoto University School of Public Health, Kyoto, Japan; 2 Department of Social Medicine, National Research Institute for Child Health and Development, Tokyo, Japan; Aichi Cancer Center Research Institute, JAPAN

## Abstract

**Background:**

Birth outcomes, such as preterm birth, low birth weight (LBW), and small for gestational age (SGA), are crucial indicators of child development and health.

**Purpose:**

To evaluate whether home visits from public health nurses for high-risk pregnant women prevent adverse birth outcomes.

**Methods:**

In this quasi-experimental cohort study in Kyoto city, Japan, high-risk pregnant women were defined as teenage girls (range 14–19 years old), women with a twin pregnancy, women who registered their pregnancy late, had a physical or mental illness, were of single marital status, non-Japanese women who were not fluent in Japanese, or elderly primiparas. We collected data from all high-risk pregnant women at pregnancy registration interviews held at a public health centers between 1 July 2011 and 30 June 2012, as well as birth outcomes when delivered from the Maternal and Child Health Handbook (N = 964), which is a record of prenatal check-ups, delivery, child development and vaccinations. Of these women, 622 women were selected based on the home-visit program propensity score-matched sample (pair of N = 311) and included in the analysis. Data were analyzed between January and June 2014.

**Results:**

In the propensity score-matched sample, women who received the home-visit program had lower odds of preterm birth (odds ratio [OR], 0.62; 95% confidence interval [CI], 0.39 to 0.98) and showed a 0.55-week difference in gestational age (95% CI: 0.18 to 0.92) compared to the matched controlled sample. Although the program did not prevent LBW and SGA, children born to mothers who received the program showed an increase in birth weight by 107.8 g (95% CI: 27.0 to 188.5).

**Conclusion:**

Home visits by public health nurses for high-risk pregnant women in Japan might be effective in preventing preterm birth, but not SGA.

## Introduction

Adverse birth outcomes, such as preterm birth, low birth weight (LBW), and small for gestational age (SGA), can have a long-term impact on child development and health [1–6]. Adverse birth outcomes are a known risk factor for maternal mental health and child maltreatment [7–9]. In Japan, like other developed countries [10,11], the proportion of preterm birth (5.8%) and LBW (boys: 8.5%, girls: 10.7%) has increased over the past three decades [11–14]. The causes of preterm birth or LBW have been considered multifactorial [15], and include, for example, maternal infection during pregnancy [16], smoking [17], low maternal BMI [18], maternal depression [19,20], lack of social support [21], maternal disease [22], and social disadvantage[23–25]. To prevent adverse health outcomes, a comprehensive intervention approach is needed because these risk factors are likely to be co-occurring.

Implementation of a home-visit program during pregnancy is a comprehensive strategy to prevent adverse birth outcomes [26,27]. Although the exact mechanism of this approach is not well clarified, many previous studies have suggested that providing tangible in-home or one-on-one psychosocial support, and improving linkages to medical providers, social services and nutrition support can encourage healthy prenatal behaviors[28–31]. However, previous randomized controlled trials (RCTs) of home-visit programs and pregnancy outcomes showed inconsistent results [32–35]. For example, Lee and colleagues [36] found that home visits before 30 weeks’ gestation for women (Black and Hispanic: 65%; under 18 years old: 24.6%) were effective for preventing LBW (5.1% versus 9.8%; p = 0.022), however McLaughlin and colleagues [37] found home visits for women (Black women: 35%, mean age: 21.8 years old) showed no significant effect in reducing LBW incidence. This is likely due to several factors, including differences in the characteristics of target participants and methods of program delivery, the reluctance of high-risk women to participate, and variation in the timing of home-visit implementation between trials [38,39]. Therefore, an assessment of the effectiveness of home visits for a wide range of high-risk women, and the timing of implementation, is needed.

Japan has a unique data collection and prenatal support system that was first established by the Maternal and Child Health (MCH) Act and MCH Law in 1965 for the promotion of maternal, newborn, and infant health. The Act promotes continuity of care through the MCH Handbook [40], which is provided for free to expectant mothers who submit a notice of pregnancy to their local government office. Women in Japan are supposed to register their pregnancy within the 11th gestational week [41]. The Handbook unifies maternal and child health into one resource, serving as a maternal health record during pregnancy and a child health record from 0–6 years, which parents can keep and take with them to appointments. In addition to the MCH Handbook, which has almost 100% coverage [40] for expectant mothers, the Act also provides health guidance to pregnant and postpartum women, and health check-ups for newborns and infants at local government health centers.

In July 2011, Kyoto city in Japan established the population-based home-visit program for all high-risk pregnant women. High-risk pregnant women were defined as teenage girls (range 14–19 years old), women with a twin pregnancy, women who registered their pregnancy late, had a physical or mental diseases, were of single marital status, non-Japanese women who were not fluent in Japanese, or elderly primiparas. At the time of pregnancy registration at the public health center, public health nurses assessed the risk level of pregnant women by conducting an interview in person using a registration questionnaire. As some women receive the home-visit program and others do not, we were provided with the opportunity to conduct a quasi-experimental study on the effectiveness of the home-visit program. As the baseline information is known at registration, the propensity of receiving the home-visit program can be assessed.

The objective of this study is to evaluate the effectiveness of the home-visit program conducted by public health nurses to high-risk pregnant women to prevent adverse birth outcomes (preterm, LBW, and SGA) by using the propensity score-matching model. We also investigated whether timing of program implementation had an effect on adverse birth outcomes.

## Methods

### Ethics statement

We used secondary administrative data from Kyoto city government in this study, which did not contain identifying information about individuals. In addition, public health nurses obtained written informed consent from pregnant women. We obtained written informed consent from Kyoto city government’s ethical committee to use this secondary administrative data which did not contain identifying information about individuals, and published an announcement about this study on the official homepage of Kyoto city government. The announcement stated that if individuals who met the inclusion criteria did not want their own data to be used, even though it was anonymised, they could request for their data to be omitted by calling the Kyoto city government. However, no one requested for their data to be omitted from the study. The study was approved by the Kyoto University Graduate School and Faculty of Medicine Ethics Committee (E1833).

### Study design and population

This was a quasi-experimental cohort study using administrative data collected in Kyoto, Japan. Data was obtained from the Department of Child and Maternal Health in Kyoto, including the baseline questionnaire conducted at pregnancy registration in public health centers, MCH handbook data, which includes data from prenatal checkups, delivery, child development, vaccinations, and home-visits. The population of Kyoto is around 1,467,000. During the period of 1 July 2011 to 30 June 2012, 11,749 women registered their pregnancy at public health centers in Kyoto. Trained public health nurses conducted interviews in person at the public health center using unified standard questionnaires to assess high-risk pregnancy. Target participants of our study were all high-risk pregnant women who registered their pregnancy in Kyoto city. High-risk pregnant women were administratively defined as follows: 1) women who had past or current physical or mental illness; 2) primiparas under the age of 20; 3) primiparas over the age of 35 with some unfavorable conditions such as poverty; 4) women who were pregnant with twins; 5) women who were late to register their pregnancy (i.e. after the 22^nd^ week of gestation) or women who were unhappy about being pregnant; 6) women with single marital status (unmarried or divorced); 7) non-Japanese women who were not fluent in Japanese, and 8) women who were assessed by public health nurses at registration as requiring any additional support including both medical, psycho-social, nutrition counseling.

**Fig 1:**
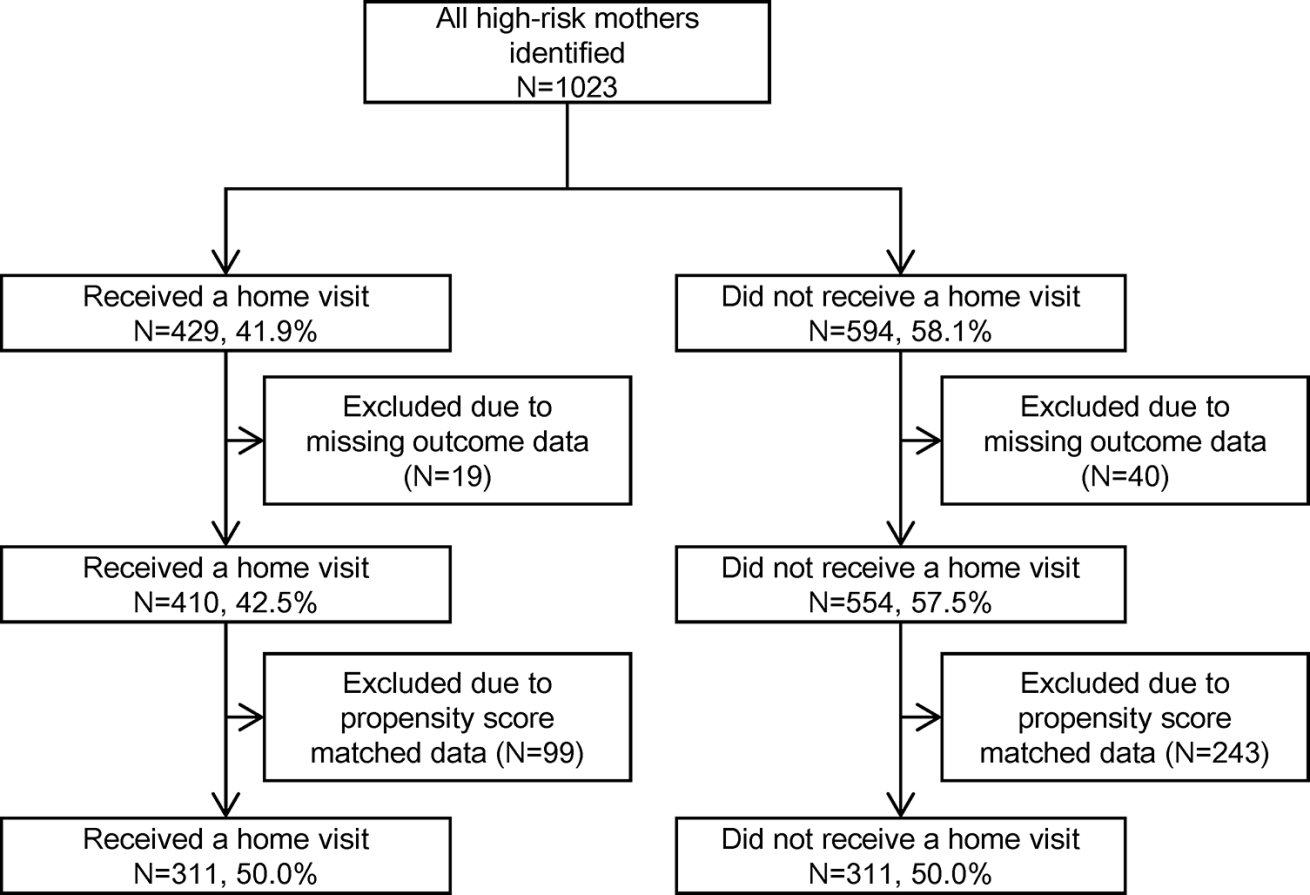
Participant flowchart.

The target population of high-risk prenatal mothers in this project was 1,023 women, and all data were used in the initial analysis to calculate propensity scores for receiving the home-visit program. That is, all high-risk pregnant women were supposed to receive home visits from public health nurses; however, 594 women (58.1%) did not receive any visits because they were not reachable. This group was used as a control sample (Fig 1).

### Home-visit programs for pregnant women in Kyoto

In this program, trained public health nurses were to make at least 1 home visit to high-risk pregnant women lasting for more than 1 hour during mid- or late-term pregnancy (mean gestational age: 27.2 (SD = 6.9) weeks, range: 7–40 weeks). Nurses received specific training about home visiting about once a year and were expected to consult with supervisors about difficult cases. The contents of the home visit were as follows: 1) checking women’s social support status and linking them to other services in the community, if needed; 2) providing information about appropriate nutrition during pregnancy, prenatal care, dental care, and child care, and 3) asking women about their physical or psychological health and linking them to medical facilities if needed. If nurses concluded that the women required more support, they provided follow-up support by phone, made another home visit, or introduced women to further social services support. Detailed components of the home visit are shown in Table 1.

**Table 1:** Key components of the home-visit program.

Component	Implementation rates (%)
Assessment of parenting support performed after birth	97.4
Information about home-visit program distributed after birth	95.2
Information about preparation for child-rearing	94.7
Consultation about health-checkups during pregnancy	90.0
Consultation about anxieties and worries about parenting	89.7
Consultation about abnormal signs during pregnancy	86.4
Referral to other institution for parenting support	85.9
Information about parenting classes	83.7
Information about preventing child accidents	66.0
Information about mother’s dental check-ups during pregnancy	65.5
Information about nutrition seminars	39.4
Other	62.7

### Outcomes

Birth outcomes, birth weight (crude value) and gestational age were obtained from birth records in the MCH handbook. Z-scores of birth weight (ZBW) were calculated and we adjusted for gestational age, sex, and parity [42]. Binary indicators were low birth weight, defined as less than 2500g; preterm birth, defined as less than 37 weeks, and small for gestational age, defined as <10 percentile.

### Covariates and independent variables

The following covariates were obtained from baseline questionnaires and interviews from trained public health nurses at the time of pregnancy registration: maternal age, paternal age, employment status during pregnancy, living with family members, marital status, parity, history of miscarriage and stillbirth, experiences of fertility treatment, late submission of pregnancy registration (over 22 weeks), twin pregnancy, maternal smoking or alcohol consumption, smoking among women’s family members, maternal physical or mental illness, limited Japanese ability among non-Japanese women, unintended pregnancy, worries about child-rearing, finances, and relationship with partner, having someone to consult with on child care, experiences of good relationships with parents, subjective economic status, maternal and paternal attitudes to child-rearing, and experiences of child-rearing. Categorical variables were used for childcare services (“Yes”, “Rarely”, or “Never”) and knowing someone with experience of child-rearing (“Yes, many people”, “Yes, a few people”, or “No, do not know anyone”). The remaining variables were binominal (see Table 2).

**Table 2:** Baseline characteristics by home-visit group and no home-visit group.

		Whole sample, N (%)			Propensity score-matched sample, N (%)		
Characteristics		HOME-VISIT PROGRAM (n = 410)	NO HOME-VISIT PROGRAM (n = 554)	*P Value*	HOME-VISIT PROGRAM (n = 311)	NO HOME-VISIT PROGRAM (n = 311)	*P Value*
Mother	Age, mean (SD), y	30.5 (7.2)	30.0 (7.0)	0.23	30.2 (7.1)	30.1 (7.2)	0.88
	Marital status						
	Married	296 (72.2)	357 (64.4)		215 (69.1)	214 (68.8)	
	Single	114 (27.8)	197 (35.6)	0.01	96 (30.9)	97 (31.2)	0.93
	Parity						
	0	333 (81.2)	313 (56.5)		235 (75.6)	233 (74.9)	
	1≥	77 (18.8)	241 (43.5)	<0.001	76 (24.4)	78 (25.1)	0.85
	History of miscarriage	94 (22.9)	119 (21.5)	0.59	62 (19.9)	67 (21.5)	0.62
	History of stillbirth	6 (1.5)	26 (4.7)	<0.001	6 (1.9)	6 (1.9)	0.99
	Late submission of notification of pregnancy (over 22 gestational weeks)	17 (4.2)	38 (6.9)	0.07	14 (4.5)	12 (3.9)	0.69
	Twin pregnancy	45 (11.0)	66 (11.9)	0.65	39 (12.5)	40 (12.9)	0.90
	Prenatal smoking	46 (11.2)	111 (20.0)	<0.001	40 (12.9)	45 (14.5)	0.56
	Prenatal alcohol consumption	41 (10.0)	67 (12.1)	0.001	33 (10.6)	34 (10.9)	0.99
	Past or present disease^a^	163 (39.8)	142 (25.6)	<0.001	104 (33.4)	104 (33.4)	0.99
	Present mental illness^b^	54 (13.2)	59 (10.7)	0.23	38 (12.2)	43 (13.8)	0.55
	Present physical disease^c^	30 (7.3)	30 (5.4)	0.23	21 (6.8)	20 (6.4)	0.87
	Foreign nationality^d^	29 (7.1)	27 (4.9)	0.15	17 (5.5)	15 (4.8)	0.72
	Unhappy about pregnancy	51 (12.4)	111 (20.0)	<0.001	40 (12.9)	42 (13.5)	0.80
	History of fertility treatment	77 (18.8)	65 (11.7)	0.002	52 (16.7)	51 (16.4)	0.99
	Having someone who can advise on child-rearing						
	Yes	407 (99.3)	547 (98.7)	0.42	308 (99.0)	307 (98.7)	0.70
	Partner	275 (67.1)	356 (64.3)	0.36	211 (71.1)	219 (70.4)	0.86
	Parents	276 (67.3)	372 (67.2)	0.96	225 (72.4)	220 (70.7)	0.66
	Step-parents	129 (31.5)	142 (25.6)	0.05	100 (32.2)	95 (30.6)	0.67
	Siblings	117 (28.5)	172 (31.1)	0.40	99 (31.8)	88 (28.3)	0.34
	Friends	319 (77.8)	374 (67.5)	<0.001	234 (75.2)	237 (76.2)	0.78
	Having someone who can give support with child-rearing						
	Yes	407 (99.3)	525 (94.8)	<0.001	308 (99.0)	307 (98.7)	0.70
	Partner	289 (70.5)	282 (50.9)	<0.001	206 (66.2)	206 (66.2)	0.99
	Parents	253 (61.7)	327 (59.0)	0.40	205 (65.9)	204 (65.6)	0.93
	Step-parents	94 (22.9)	103 (18.6)	0.10	72 (23.2)	72 (23.2)	0.99
	Siblings	58 (14.2)	92 (16.6)	0.30	46 (14.8)	44 (14.2)	0.82
	Friends	45 (11.0)	44 (7.9)	0.11	31 (10.0)	29 (9.3)	0.79
	Working at time of pregnancy registration	195 (47.6)	275 (49.6)	0.67	164 (52.7)	154 (49.5)	0.72
	Returning to hometown before delivery (‘*satogaeri*’^e^)	178 (43.4)	204 (36.8)	0.04	138 (44.4)	132 (42.4)	0.63
	Used childcare services						
	Yes	291 (71.0)	446 (80.5)		233 (74.9)	239 (76.9)	
	Rarely	80 (19.5)	73 (13.2)	<0.001	60 (19.3)	56 (18.0)	
	Never	27 (2.9)	12 (2.2)		11 (3.5)	11 (3.5)	0.91
	Felt unloved by parents when growing up	35 (8.5)	49 (8.8)	0.18	25 (8.0)	26 (8.4)	0.97
	Knows someone with experience in child-rearing						
	Yes, many people	264 (64.4)	407 (73.5)		210 (67.5)	225 (72.4)	
	Yes, a few people	80 (19.5)	76 (13.7)	<0.001	60 (19.3)	51 (16.4)	
	No, do not know anyone	56 (13.7)	46 (8.3)		35 (11.3)	31 (10.0)	0.60
	Low capacity of child rearing	8 (2.0)	28 (5.1)	0.01	7 (2.3)	8 (2.6)	0.79
	Worried about pregnancy due to previous negative experiences of delivery	12 (2.9)	28 (5.1)	0.10	11 (3.5)	7 (2.3)	0.34
	Worried about:						
	Child-rearing	192 (46.8)	213 (38.5)	0.01	142 (45.7)	140 (45.0)	0.87
	Money	184 (44.9)	250 (45.1)	0.94	142 (45.7)	146 (47.0)	0.75
	Disease	89 (21.7)	97 (17.5)	0.10	62 (19.9)	63 (20.3)	0.92
	Partner	28 (6.8)	52 (9.4)	0.16	24 (7.7)	28 (9.0)	0.56
	Lack of support or advice	35 (8.5)	34 (6.1)	0.15	22 (7.1)	26 (8.4)	0.55
	Job	99 (24.2)	130 (23.5)	0.81	80 (25.7)	77 (24.8)	0.78
	Relationships with neighbors	64 (15.6)	62 (11.2)	0.04	45 (14.5)	47 (15.1)	0.82
	Relationships with relatives	16 (3.9)	37 (6.7)	0.06	15 (4.8)	18 (5.8)	0.59
Partner	Age, mean (SD), y	33.9 (8.0)	32.9 (7.8)	0.07	33.4 (7.6)	33.0 (7.9)	0.55
	Informed partner about pregnancy	397 (96.8)	504 (91.0)	0.001	298 (95.8)	297 (95.5)	0.82
	Partner unhappy about pregnancy	42 (10.2)	85 (15.3)	<0.001	30 (9.7)	32 (10.3)	0.97
Household	Living with:						
	Partner	292 (71.2)	330 (59.6)	<0.001	210 (67.5)	206 (66.2)	0.93
	Partner's parents	107 (26.1)	158 (28.5)	0.41	86 (27.7)	93 (29.9)	0.54
	Family members smoked during pregnancy	150 (36.6)	212 (38.3)	0.59	116 (37.3)	116 (37.3)	0.99
	Economic problems	112 (27.3)	123 (22.2)	0.07	72 (23.2)	73 (23.5)	0.92

Note from questionnaire

^a^"Do you have any disease that is currently under treatment or was treated in the past?"

^b^ From risk assessment by public health nurses in first interview.

^c^ From risk assessment by public health nurses in first interview.

^d^ Prenatal mothers who emigrated to Japan or were staying in Japan long-term.

^e^ ‘*satogaeri’*: a tradition in which pregnant women return to the family home prior to delivery to stay with their parents for support before and after childbirth.

### Statistical analysis

Of the 1,023 baseline samples, we excluded participants who had missing birth-outcome data (home-visit program: n = 19, no home-visit program: n = 40, 5.8% in total participants) from the analysis (total N = 964 women, home-visit program: n = 410, no home-visit program: n = 554). Then, propensity score-matched analysis was performed to reduce potential bias on receiving the home-visit program. The probability of home-visit participation was estimated by all baseline characteristics using logistic regression, because these characteristics possibly associate with participation in the home-visit program. Propensity-score matching was performed by using the following algorithm: 1:1 nearest-neighbor match method with a caliper of 0.4 SD and no replacement. Finally, 311 women who received the home-visit program and 311 women who did not were included in the analysis (that is, N = 622). Variables used to estimate the propensity to participate in the program were sufficiently high, with C-statistics at 0.77. No significant difference was observed between the baseline characteristics of the home-visit program group and the no home-visit group (Table 2, right columns). The propensity-matched pairs were compared using logistic regression analysis, and multivariate regression analysis after adjusting for all baseline variables. In addition, sub-group analysis was performed for the timing of home-visit implementation, which was divided into two subgroups (home visits at <28 gestational weeks, home visits at ≥28 weeks). Missing data of covariates such as alcohol consumption (n = 4), experiences of fertility treatment (n = 12), physical or mental illness (n = 2), employment status (n = 25), living with family members (n = 162), experiences of good relationships with parents (n = 25), maternal and paternal attitudes of child rearing (n = 32) and experiences of child-rearing (n = 12) were treated as dummy variables. Where paternal age was missing in the data, the mean age was imputed. Stata version 13 was used to perform the analysis between January 2014 and June 2014.

## Results

Table 2 shows the baseline characteristics of prenatal mothers at pregnancy registration in the home-visit group (n = 410) and the no home-visit group (n = 554) before propensity-score matching. Mean gestational age for infants of mothers in the home-visit group was 27.2 (SD = 6.9) weeks. Pregnant women who received home visits were more likely to be experiencing their first pregnancy (n = 333, 81.2%), diagnosed with a disease (n = 163, 39.8%), and worried about child-rearing (n = 192, 46.8%) or relationships with neighbors (n = 64, 15.6%) compared with women who did not receive home visits. Pregnant women who did not receive home visits were more likely to smoke (n = 111, 20.0%), drink alcohol (n = 67, 12.1%), be unmarried (n = 197, 35.6%), feel unhappy about their pregnancy (n = 111, 20.0%), or had partners who were unhappy about their pregnancy (n = 85, 15.3%) compared with women in the home-visit group. After performing propensity-score matching with the comparison group, no significant difference was observed between variables (see Table 2).

Table 3 shows the birth outcomes before and after propensity-score matching. Before propensity-score matching, women from the home-visit group had a heavier birth weight (2905.3 g, SD = 499.5 g), longer gestational age (38.7 weeks, SD = 1.8 weeks), higher ZBW (-0.04, SD = 1.1), less LBW infants (n = 85, 19.2%), less preterm birth (n = 40, 9.8%), and less SGA infants (n = 52, 11.7%) compared to participants who did not receive the home-visit program. After propensity-score matching, women from the home-visit group had a heavier birth weight (2933.3 g, SD = 473.4 g), longer gestational age (38.6 weeks, SD = 1.8 weeks), and less preterm birth (n = 34, 10.9%) compared to women who did not receive the home-visit program.

**Table 3:** Description of outcomes of home-visit program vs. no home-visit program.

	Whole sample, N (%)			Propensity score-matched sample, N (%)		
Birth outcomes	HOME-VISIT PROGRAM (mother: n = 410, child: n = 443)	NO HOME-VISIT PROGRAM (mother: n = 554, child: n = 625)	*P Value*	HOME-VISIT PROGRAM (mother: n = 311, child: n = 311)	NO HOME-VISIT PROGRAM (mother: n = 311, child: n = 311)	*P Value*
Birth weight, mean (SD), g	2905.3 (499.5)	2767 (584.2)	<0.001	2933.3 (473.4)	2825.5 (553.1)	0.01
Gestational age, mean (SD), week	38.7 (1.8)	38.1 (2.4)	<0.001	38.6 (1.8)	38.0 (2.7)	0.003
z-score of birth weight, mean (SD),	-0.04 (1.1)	-0.29 (1.0)	<0.001	-0.03 (1.0)	-0.08 (1.0)	0.55
LBW (<2500g)	85 (19.2)	159 (25.4)	0.02	50 (16.1)	65 (20.9)	0.12
Preterm birth (<37 weeks)	40 (9.8)	78 (14.1)	0.04	34 (10.9)	52 (16.7)	0.04
SGA (10 percentile<)	52 (11.7)	110 (17.6)	<0.001	30 (9.7)	31 (10.0)	0.89
Sex (male)	229 (51.7)	325 (52.0)	0.92	160 (51.5)	173 (55.6)	0.30
Timing of home visit during pregnancy						
Gestational age, mean (SD), week	27.2 (6.9)			27.1 (7.0)		
28 weeks<, n (mean, SD)	191 (21.3, 4.73)			145 (21.0, 4.88)		
28 weeks≥, n (mean, SD)	209 (32.7, 2.93)			159 (32.6, 3.00)		
Missing data, n	10			7		

Abbreviations: LBW: low birth weight; SGA: small for gestational age.

Table 4 shows the coefficient and odds ratios (ORs) of the home-visit program for birth outcomes. Before propensity-score matching was conducted in the univariate model, women in the home-visit program during their pregnancy had a significantly heavier birth weight (coefficient: 138.3g, 95% confidence interval [CI]: 63.2 to 213.4), longer gestational age (coefficient: 0.67 week, 95% CI: 0.33 to 1.00), higher ZBW (coefficient: 0.25, 95% CI: 0.11 to 0.38), LBW (OR: 0.70, 95% CI: 0.49 to 0.98), preterm birth (OR: 0.62, 95% CI: 0.41 to 0.94), SGA (OR: 0.62, 95% CI: 0.43 to 0.91) than women who did not receive the home-visit program. These associations remained significant in the multivariate adjusted model, except for ZBW and SGA. Further, in the propensity-matched sample for the home-visit group, a heavier birth weight, longer gestational age, and lower odds of preterm birth remained significant during pregnancy, although odds of LBW became non-significant. In the final model—the multivariate adjustment of the propensity-score matched sample—pregnant women in the home-visit group delivered infants with a heavier birth weight of 99.1g (95% CI: 20.5 to 177.6) and a longer gestational age of 0.61 weeks (95% CI: 0.25 to 0.96), and were 74% less likely to deliver preterm, compared to pregnant women who did not receive home visits.

**Table 4:** Effects of home-visit program on birth outcomes.

	Birthweight	Gestational age	Z-score of birth weight	LBW (<2500g)	Preterm birth (<37 week)	SGA (10^th^ percentile<)
Model	Coefficient (95% CI)			Odds ratio (95% CI)		
Unadjusted	**138.3 (63.2 to 213.4)**	**0.67 (0.33 to 1.00)**	**0.25 (0.11 to 0.38)**	**0.70 (0.49 to 0.98)**	**0.62 (0.41 to 0.94)**	**0.62 (0.43 to 0.91)**
Multivariable adjusted	**115.4 (51.2 to 179.7)**	**0.49 (0.19 to 0.79)**	0.86 (-0.45 to 0.22)	**0.63 (0.40 to 0.99)**	**0.47 (0.26 to 0.84)**	0.81 (0.97 to 1.07)
Propensity score-matched	**107.8 (27.0 to 188.5)**	**0.55 (0.18 to 0.92)**	0.47 (-0.11 to 0.20)	0.73 (0.48 to 1.09)	**0.62 (0.39 to 0.98)**	0.96 (0.56 to 1.66)
Propensity score-matched + multivariable adjusted	**99.1 (20.5 to 177.6)**	**0.61 (0.25 to 0.96)**	0.01 (-0.14 to 0.17)	0.26 (0.052 to 1.26)	**0.26 (0.001 to 0.64)**	0.71 (0.22 to 2.34)

Abbreviations: CI: confidence interval; LBW: low birth weight; SGA: small for gestational age. Bold value signifies p<0.05.

Table 5 shows the subgroup analyses of the effectiveness of the home-visit program by timing of implementation (i.e. whether the program was implemented before or after 28 weeks’ gestation). In the propensity-score matched sample, women who entered the home-visit program late (after 28 gestational weeks, n = 159) showed a longer gestational age (coefficient: 0.65 weeks, 95% CI: 0.09 to 1.20) compared to women who did not receive home visits. Further, a marginal protective effect on preterm birth was found among women who entered the program late compared to women who were registered in the program earlier (OR: 0.57, 95% CI: 0.31 to 1.06). However, early implementation of the home-visit program (before 28 gestational weeks, n = 145) failed to show longer gestational age nor a protective effect on preterm than women in the comparison group, suggesting that joining the home-visit program after 28 weeks’ gestation was more effective to achieve longer gestational age and to be protective for preterm.

**Table 5:** Subgroup analysis on timing of home-visit program implementation.

	Birth weight	Gestational age	Z-score of birth weight	LBW (<2500g)	Preterm birth (<37 weeks)	SGA (10 percentile<)
Model	Coefficient (95% CI)			Odds ratio (95% CI)		
28 weeks<						
Unadjusted (n = 191)^a^	**115.2 (17.2 to 213.2)**	**0.48 (0.68 to 0.89)**	**0.23 (0.05 to 0.40)**	0.86 (0.56 to 1.32)	0.70 (0.41 to 1.19)	0.78 (0.48 to 1.25)
Multivariable adjusted (n = 191)^a^	**156.4 (74.7 to 238.2)**	**0.66 (0.27 to 1.05)**	0.09 (-0.07 to 0.25)	0.63 (0.36 to 1.09)	**0.35 (0.16 to 0.76)**	1.00 (0.54 to 1.88)
Propensity score-matched (pair of 145)	107.3 (-4.0 to 218.7)	0.37 (-0.13 to 0.88)	0.10 (-0.12 to 0.33)	0.73 (0.40 to 1.32)	0.72 (0.35 to 1.47)	0.87 (0.41 to 1.82)
28 weeks> =						
Unadjusted (n = 209)^a^	**156.4 (67.8 to 245.0)**	**0.83 (0.44 to 1.21)**	**0.27 (0.09 to 0.44)**	**0.57 (0.35 to 0.91)**	0.58 (0.34 to 1.01)	**0.46 (0.28 to 0.76)**
Multivariable adjusted (n = 209)^a^	**92.6 (13.6 to 171.5)**	**0.40 (0.06 to 0.74)**	0.09 (-0.07 to 0.26)	0.65 (0.37 to 1.13)	0.60 (0.30 to 1.22)	0.64 (0.34 to 1.20)
Propensity score-matched (pair of 159)	104.0 (-15.8 to 223.9)	**0.65 (0.09 to 1.20)**	0.03 (-0.19 to 0.25)	0.71 (0.40 to 1.26)	0.57^b^ (0.31 to 1.06)	0.91 (0.39 to 2.14)

^a^ no home visit (n = 554)

^b^ *p =* 0.07.

Abbreviations: CI: confidence interval; LBW: low birthweight; SGA: small for gestational age. Bold value signifies p<0.05.

## Discussion

The present study evaluated the effectiveness of the home-visit program for high-risk pregnant women in Japan on birth outcomes (birth weight, gestational age, Z-scores of birth weight) using a propensity-score matched sample. We found that home visits from trained public health nurses at least once during pregnancy were effective to prevent preterm birth, but not small for gestational age among high-risk pregnant women in Japan.

To our knowledge, this is the first study to investigate the effectiveness of the home-visit program for a wide range of high-risk pregnant women as a public healthcare measure. However, two randomized controlled trials of home-visit programs [36,37], which investigated the efficacy of birth outcomes, suggested that the effectiveness of such programs is still inconsistent, while two recent observational studies conducted in the United States (US) using propensity score-matched analysis found home-visit programs to be effective [33,43]. Both US studies concluded that participating in the home-visit program reduced the risk of adverse birth outcomes in disadvantaged populations (i.e. people who received Medicaid). Our finding is consistent with these previous studies in showing the effectiveness of the home-visit program in preventing adverse birth outcomes, although the definition of disadvantaged population is different (i.e. our definition of ‘high-risk pregnant women’ did not only focus on economic status but on medical conditions, social disadvantages and other factors).

In the Japanese healthcare system, all pregnant women are screened by public health nurses at registration of their pregnancy, or within 11 weeks’ gestation. In addition, high-risk women receive comprehensive support and are referred to appropriate follow-up services[44]. This system enables high-risk women to be followed-up from the prenatal to postnatal period, and facilitates the first contact between public health services and high-risk pregnant women. Some components of the home-visit program, such as consultations on maternal anxiety, nutrition education and health-check ups, might improve birth weight. Although we tried to provide the home-visit program for all high-risk pregnant women, approximately half of the high-risk women (n = 554) were not reachable and showed worse risk factors, such as smoking and drinking alcohol, for poor birth outcomes. It is another challenge to support the super-high-risk pregnant women who were not reachable in general health care system.

We found that late admission to the home-visit program was effective to prevent preterm birth, although early admission to the home-visit program did not. The reason for this difference is unknown. It is possible that perinatal care or advice from public health nurses might be more effective closer to delivery. Further study is needed to elucidate the mechanism on why receiving home visits later in pregnancy is more effective to prevent preterm birth.

This study has some limitations. First, this study was of a quasi-experimental cohort design, so there may be unobserved variables and unknown confounding factors, such as maternal personality or characteristics. These factors may affect birth outcomes despite adjusting for baseline variables using the propensity score. Second, potential selection bias exists because approximately 5.8% (59/1023) of the data was omitted due to missing outcome data. However, there were no significant differences in the baseline characteristics between analyzed and omitted samples, so the present results represent the overall sample data. Finally, we could not count the dosage of the program and the number of times nurses visited participants’ homes. Further study is needed to investigate the association between outcomes and dosage of the program.

Despite these limitations, we found that the home-visit program for high-risk pregnant women by public health nurses significantly prevented preterm birth. Further studies with larger sample sizes are needed to measure the timing and dosage of the intervention in order to clarify the dose-response relationship in the home-visit program.

## Conclusion

Our findings suggest that home visits by public health nurses for high-risk pregnant women in Japan might be effective in preventing preterm birth, but not SGA. This study adds to the evidence of the effectiveness of population-based home-visit programs as a public healthcare measure.
