# Correction: Effectiveness of Home Visits in Pregnancy as a Public Health Measure to Improve Birth Outcomes

**DOI:** 10.1371/journal.pone.0152354

**Published:** 2016-03-21

**Authors:** Kayoko Ichikawa, Takeo Fujiwara, Takeo Nakayama

There are a number of errors in Table 2. Please see the corrected Table 2 here.

**Table 2:** Baseline characteristics and outcomes of the home-visit program

Characteristics		Whole sample			Propensity score-matched sample		
		N. (%)			N. (%)		
		HOME VISIT PROGRAM(+) (n = 410)	HOME VISIT PROGRAM(-) (n = 554)	*P Value*	HOME VISIT PROGRAM(+) (n = 311)	HOME VISIT PROGRAM(-) (n = 311)	*P Value*
Mother	Age, mean (SD), y	30.5 (7.2)	30.0 (7.0)	0.23	30.2 (7.1)	30.1 (7.2)	0.88
	Marital status						
	Married	296 (72.2)	357 (64.4)		215 (69.1)	214 (68.8)	
	Unmarried	114 (27.8)	197 (35.6)	0.01	96 (30.9)	97 (31.2)	0.93
	Parity						
	0	333 (81.2)	313 (56.5)		235 (75.6)	233 (74.9)	
	1≥	77 (18.8)	241 (43.5)	<0.001	76 (24.4)	78 (25.1)	0.85
	History of miscarriage	94 (22.9)	119 (21.5)	0.59	62 (19.9)	67 (21.5)	0.62
	History of stillbirth	6 (1.5)	26 (4.7)	<0.001	6 (1.9)	6 (1.9)	0.99
	Late submission of notification of pregnancy (over 22week)	17 (4.2)	38 (6.9)	0.07	14 (4.5)	12 (3.9)	0.69
	Twin pregnancy	45 (11.0)	66 (11.9)	0.65	39 (12.5)	40 (12.9)	0.90
	Prenatal smoking	46 (11.2)	111 (20.0)	<0.001	40 (12.9)	45 (14.5)	0.56
	Prenatal alcohol consumption	41 (10.1)	67 (12.6)	0.23	33 (10.7)	34 (11.0)	0.90
	Past or present disease*	163 (40.0)	142 (26.1)	<0.001	104 (33.6)	104 (33.6)	1.00
	Present mental illness†	54 (13.2)	59 (10.7)	0.23	38 (12.2)	43 (13.8)	0.55
	Present physical disease‡	59 (14.4)	73 (13.2)	0.59	21 (6.8)	20 (6.4)	0.87
	Foreign nationality§	29 (7.1)	27 (4.9)	0.15	17 (5.5)	15 (4.8)	0.72
	Unhappy about pregnancy	51 (12.6)	111 (20.9)	0.001	40 (13.1)	42 (13.7)	0.84
	History of fertility treatment	77 (19.2)	65 (12.2)	0.004	52 (17.1)	51 (16.7)	0.91
	Having someone who can give advice on child rearing						
	Yes	407 (99.3)	525 (94.8)	0.42	308 (99.0)	307 (98.7)	0.70
	Partner	275 (67.1)	356 (64.3)	0.36	211 (71.1)	219 (70.4)	0.86
	Parents	276 (67.3)	372 (67.2)	0.96	225 (72.4)	220 (70.7)	0.66
	Step-parents	129 (31.5)	142 (25.6)	0.05	100 (32.2)	95 (30.6)	0.67
	Siblings	117 (28.5)	172 (31.1)	0.40	99 (31.8)	88 (28.3)	0.34
	Friends	319 (77.8)	374 (67.5)	<0.001	234 (75.2)	237 (76.2)	0.78
	Having someone who can give support with child rearing						
	Yes	407 (99.3)	525 (94.8)	<0.001	308 (99.0)	307 (98.7)	0.70
	Partner	289 (70.5)	282 (50.9)	<0.001	206 (66.2)	206 (66.2)	0.99
	Parents	253 (61.7)	327 (59.0)	0.40	205 (65.9)	204 (65.6)	0.93
	Step-parents	94 (22.9)	103 (18.6)	0.10	72 (23.2)	72 (23.2)	0.99
	Siblings	58 (14.2)	92 (16.6)	0.30	46 (14.8)	44 (14.2)	0.82
	Friends	45 (11.0)	44 (7.9)	0.11	31 (10.0)	29 (9.3)	0.79
	Working at time of pregnancy registration	195 (50.4)	275 (53.0)	0.44	164 (4.9)	154 (51.7)	0.44
	Returning to hometown before deliver ‘Satogaeri’	178 (43.4)	204 (36.8)	0.04	138 (44.4)	132 (42.4)	0.63
	Used child care services						
	Yes	291 (73.1)	446 (84.0)		233 (76.6)	239 (78.1)	
	Rarely	80 (20.1)	73 (13.8)	<0.001	60 (19.7)	56 (18.3)	
	Never	27 (6.8)	12 (2.3)		11 (3.6)	11 (3.6)	0.90
	Feeling unloved by parents when growing up	35 (8.9)	49 (9.5)	0.76	25 (8.4)	26 (8.7)	0.89
	Knowing someone who has experience in rearing a child						
	Yes, many people	264 (66.0)	407 (76.9)		210 (68.9)	225 (72.3)	
	Yes, a few people	80 (20.0)	76 (14.4)	0.001	60 (19.7)	51 (16.6)	
	No, do not know	56 (14.0)	46 (8.7)		35 (11.5)	31 (10.1)	0.48
	Low capacity of child rearing	8 (2.0)	28 (5.1)	0.01	7 (2.3)	8 (2.6)	0.79
	Worrying about pregnancy due to previous negative experiences of delivery	12 (2.9)	28 (5.1)	0.10	11 (3.5)	7 (2.3)	0.34
	Worrying about:						
	Child rearing	192 (46.8)	213 (38.5)	0.01	142 (45.7)	140 (45.0)	0.87
	Money	184 (44.9)	250 (45.1)	0.94	142 (45.7)	146 (47.0)	0.75
	Disease	89 (21.7)	97 (17.5)	0.10	62 (19.9)	63 (20.3)	0.92
	Partner	28 (6.8)	52 (9.4)	0.16	24 (7.7)	28 (9.0)	0.56
	Supporters or advisers	35 (8.5)	34 (6.1)	0.15	22 (7.1)	26 (8.4)	0.55
	Job	99 (24.2)	130 (23.5)	0.81	80 (25.7)	77 (24.8)	0.78
	Relationships with neighbors	64 (15.6)	62 (11.2)	0.04	45 (14.5)	47 (15.1)	0.82
	Relationships with relatives	16 (3.9)	37 (6.7)	0.06	15 (4.8)	18 (5.8)	0.59
Partner	Age, mean (SD), y	33.9 (8.0)	32.9 (7.8)	0.07	33.4 (7.6)	33.0 (7.9)	0.55
	Knowing about partner's pregnancy	397 (98.0)	504 (96.0)	0.08	298 (97.4)	297 (97.7)	0.80
	Unhappy about partner’s pregnancy	42 (10.7)	85 (17.3)	0.005	30 (10.2)	32 (10.9)	0.79
Family	Living with:						
	Partner	292 (92.4)	330 (83.8)	0.001	210 (90.5)	206 (90.4)	0.95
	Partner's parents	107 (26.1)	158 (28.5)	0.41	86 (27.7)	93 (29.9)	0.54
	Smoking during partner's pregnancy	150 (36.6)	212 (38.3)	0.59	116 (37.3)	116 (37.3)	0.99
	Economic problems	112 (27.3)	123 (22.2)	0.07	72 (23.2)	73 (23.5)	0.92

Note From the questionnaire: a "Do you have any disease that is currently under treatment or was treated in the past?"

b From risk assessment by public health nurses in first interview

c From risk assessment by public health nurses in first interview

d Prenatal mothers who emigrated to Japan or were staying in Japan long-term

e Abbreviations: LBW: low birth weight; SGA: small for gestational age

There are a number of errors in Table 4. Please see the corrected Table 4 here.

**Table 4:** Effects of home-visit program on birth outcomes

Model	BW ^a^	Gestational age	Z-score of birth weight	LBW ^a^ (<2500g)	Preterm birth(<37 week)	SGA ^a^ (10 percentile<)
	Coefficient (95% CI)			Odds ratio (95% CI)		
Unadjusted	**138.3**	**0.67**	**0.25**	**0.70**	**0.62**	**0.62**
	**(63.2 to 213.4)**	**(0.33 to 1.00)**	**(0.11 to 0.38)**	**(0.49 to 0.98)**	**(0.41 to 0.94)**	**(0.43 to 0.91)**
Multivariable adjusted	**115.4**	**0.49**	0.09	**0.63**	**0.47**	0.81
	**(51.2 to 179.7)**	**(0.19 to 0.79)**	(-0.05 to 0.22)	**(0.40 to 0.99)**	**(0.26 to 0.84)**	(0.50 to 1.32)
Propensity score-matched	**107.8**	**0.55**	0.47	0.73	**0.62**	0.96
	**(27.0 to 188.5)**	**(0.18 to 0.92)**	(-0.11 to 0.20)	(0.48 to 1.09)	**(0.39 to 0.98)**	(0.56 to 1.66)
Propensity score-matched + multivariable adjusted	**99.1**	**0.61**	0.01	0.26	**0.03**	0.71
	**(20.5 to 177.6)**	**(0.25 to 0.96)**	(-0.14 to 0.17)	(0.052 to 1.26)	**(0.001 to 0.64)**	(0.22 to 2.34)

Note ^a^ Abbreviations: BW: birth weight; LBW: low birth weight; SGA: small for gestational age
